# Long term WEB results – still going strong at 5 years?

**DOI:** 10.1177/15910199221139542

**Published:** 2022-11-17

**Authors:** Kemal Alpay, Alberto Nania, Rahul Raj, Jussi Numminen, Riitta Parkkola, Riitta Rautio, Jonathan Downer

**Affiliations:** 1 Department of Radiology, 60652 Turku University Hospital, Turku, Finland; 2 Department of Clinical Neurosciences, 3124 University of Edinburgh, Edinburgh, UK; 3 Department of Neurosurgery, 3836 Helsinki University Hospital and University of Helsinki, Finland; 4 Department of Radiology, 3836 Helsinki University Hospital, Helsinki, Finland

**Keywords:** WEB, aneurysm, endovascular treatment, subarachnoid hemorrhage

## Abstract

**Objective:**

The aim of our multi-center study is to examine 5-year radiological outcomes of intracranial aneurysms (IAs) treated with the Woven EndoBridge (WEB).

**Methods:**

All patients treated with WEB between January 2013 and December 2016 were included. Patient and aneurysm characteristic data was collected from the electronic patient record. Aneurysm occlusion was assessed using a 3-grade scale: complete occlusion, neck remnant, and aneurysm remnant. Complete occlusion and neck remnant were considered as adequate occlusion, whereas aneurysm remnant was assessed as an inadequate occlusion.

**Results:**

A total of 66 patients (72.7% female) with 66 IAs (n = 25 acutely ruptured) were treated with WEB. The mean age of patients was 55.6 years (range: 36–71 years). The mean width of the aneurysm neck was 4.5 mm (range: 2−9 mm). 5-year imaging follow-up data was not available for 16.6% patients (n = 11). During the follow up period, 14.5% of IAs (n = 8/55) required retreatment within 24 months of initial treatment with the WEB. A total of 55 IAs were analyzed for 5-year radiological outcome. Of these, including IAs required retreatment, 47.3% of IAs (n = 26/55) were occluded completely, 36.4% (n = 20/55) had neck remnant and 16.3% (n = 9/55) had recanalized. 83.7% of IAs were occluded adequately. None of the IAs rebled after initial treatment with WEB.

**Conclusion:**

WEB can provide acceptable adequate occlusion rates at 5 years. Furthermore, recanalization appears to be unlikely after the first two years post-treatment. The results of large studies are needed to confirm these promising long term radiological outcomes

## Introduction

In the years since the Guglielmi detachable coiling system was introduced endovascular treatment has become an option for most intracranial aneurysms (IAs), with the help of developing technology. Endovascular treatment with simple coiling for both ruptured and unruptured intracranial aneurysms has been proven to be safe and effective.^1,2^ However, the treatment of IA with simple coiling carries a 20% risk of recanalization.^3^ Particularly, large (≥10 mm) intracranial aneurysms with wide neck (≥4 mm) are prone to reopening after conventional coiling.^4^ Even though stent-assisted coiling and balloon assisted coiling provide better radiological outcomes for intracranial aneurysms than simple coiling, the rates of thromboembolic complications are not insignificant and are seen in approximately 10% of patients.^5,6^ Apart from the risk of complication with stent-assisted coiling, the need for dual anti-platelet medication is another disadvantage, particularly when treating patients with acutely ruptured intracranial aneurysms. Intraluminal flow-diverters provide a rate of 90% occlusion with low complication profile for unruptured intracranial aneurysms; however, they carry a higher risk of complications in the treatment of acutely ruptured intracranial aneurysms.^7,8^

Intrasaccular flow-diversion with the Woven Endobridge (WEB) (Microvention, California, USA) is a treatment for wide-necked IAs. The WEB is a self-expandible, retrievable and detachable nitinol braided cage. The first-generation WEB-DL (Dual layer) was launched in 2010 and was replaced with WEB-SL (single layer) and WEB-SLS (single layer sphere) in 2013^9,10^ followed by full visibility with nitinol-platinum DFT wires in 2014. A flow quantification study showed that WEB disturbs outflow more than inflow velocity, eventually causing thrombosis of the aneurysm.^11^ Several multicenter studies have demonstrated efficacy and safety of the WEB for both unruptured and ruptured IAs.^12–15^

The objective of our multi-center retrospective study was to assess radiological outcomes of aneurysms at 5 years after WEB treatment.

## Materials and methods

### Patient selection and data collection

**Figure 1.**
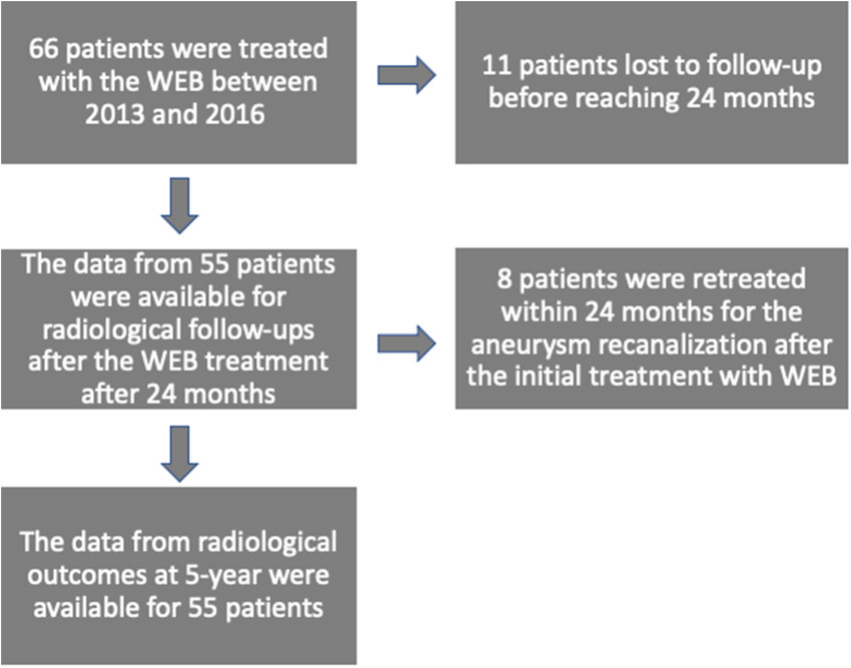
Flow chart of the population included safety and efficacy at 5 years.

We retrospectively collected the data of patients with unruptured or ruptured IAs treated with WEB between 2013 and 2016. A Flowchart of the population included in this study for safety and efficacy at 5 year is summarized in Figure 1. All procedure related symptomatic complications (i.e., transient ischemic attack and ischemic or hemorrhagic stroke) were recorded in the electronic patient file database. All morphologic features (width, height, irregular shape, and width of the neck) of the IAs were determined using 3-Dimensional rotational angiography. Thrombosis in aneurysm sacs was detected using preoperative magnetic resonance angiography (MRA) or computed tomography angiography (CTA).

All possible treatment methods for recurrent IAs (e.g., coiling, stent assisted coiling, balloon assisted coiling, microsurgical clipping) were considered and discussed at a multidisciplinary meeting. Digital subtraction angiography (DSA) was used to decide whether WEB was a feasible treatment. The IAs were considered wide-necked if the neck width was >4 mm and/or the dome-to-neck ratio was <2. WEB was selected for wide-necked bifurcation aneurysms, which were considered difficult to treat using conventional methods.

### The WEB treatment

Neurointerventions were carried out via femoral or radial access in a bi-plane angiographic suite. All patients were under general anesthesia during the interventions. The dimensions of the aneurysm were determined using 3-dimensional rotational angiography to assess the optimal size of the WEB. In accordance with the manufacturer's guidelines, the WEBs were delivered through an appropriate size VIA microcatheter (Sequent Medical, Aliso Viejo, California, USA). In cases in which an ancillary stent was inserted, dual antiplatelet therapy was initiated and typically continued for 6 weeks followed by Aspirin alone for 3 months to 1 year. In two centers VerifyNow® or Multiplate® (Accumetrics, San Diego, CA, USA) test was used to determine the platelet response to antiplatelet medication. Multiplate Adenosine Diphosphate < 30U or platelet reactivity unit <208 was considered a sufficient response.

### Radiological and clinical follow-up

All patients were scheduled for radiological follow-ups according to institutional protocols after the treatment with WEB. Initial radiological follow-up was scheduled either as a DSA or MRA between six- to twelve months post-treatment according to institutional protocols. Subsequently, the remainder of the radiological follow-up was performed using MRA or CTA at either 24 and 60 months or 60 months only. Aneurysm occlusion was assessed using a 3-grade scale: complete occlusion, neck remnant, and aneurysm remnant. Complete occlusion and neck remnant were considered as adequate occlusion, whereas aneurysm remnant was assessed as an inadequate occlusion.

### Statistical analysis

Descriptive statistics were used. Categorical variables were presented as numbers and percentages. Continuous variables were presented as the mean with ranges.

### Ethical issues

Local institutional review board waived the need for formal consent for this retrospective registry study.

## Results

### Patient and aneurysm characteristics

A total of 66 patients (72.7%, 48 females, mean age 55.6 years, range: 36–71) with 66 IAs were treated with WEB. Of which, 37.9% (n = 25) were treated in the setting of acute aSAH. The basilar tip (n = 20, 30.3%) was the most common location. Aneurysms with partially thrombosed or multilobular sac consisted of 6.1% (n = 4) and 28.8% (n = 19) of IAs, respectively. The mean maximum size of IAs was 7.9 mm (range: 4−16 mm). The mean width and height of the IAs were 6.3 mm (range: 3−13 mm) and 7.2 mm (4−13 mm), respectively. The mean width of the neck was 4.5 mm (range: 2−9 mm). Patient and aneurysm characteristics were summarized in Table 1.

**Table 1.** Patient and aneurysm characteristics.

No. of patients	66
Proportion of females	72.7%
Aneurysm Location	
Pcom	9.1% (6)
MCA	27.3% (18)
Acom	24.2% (16)
Basilar Tip	30.3% (20)
ACA	4.5% (3)
PCA	3% (2)
Vertebrobasillar	1.5% (1)
Thrombosed sac	6.1% (4)
Multilobular sac	28.8% (19)
Aneurysm Diameters	
Maximum size	7.9 (4–16 mm)
Width	6.3 (3–13 mm)
Height	7.2 (4–13 mm)
Width of the neck	4.5 (2–9 mm)
Acutely Ruptured	37.9% (25)

Pcom, posterior communicating artery; MCA, middle cerebral artery; Acom, anterior communicating artery; ACA, anterior cerebral artery; PCA, posterior cerebral artery.

### Treatment characteristics

A total of 67 second generation (WEB SL/SLS) devices were implanted in 66 IAs. Of which, 61 IAs were treated with WEB alone. The adjunctive devices used were coils in two, stents in two, and stent and coils in one.

### Missing data

The data regarding to 5-year imaging follow-up were not available for 16.6% (n = 11/66). Of which, 5 patients died (3 due to complications of aSAH before reaching any follow-up, 2 due to unrelated reasons) before reaching to the 5-year follow-up. Six patients with median follow-up time of 26 months were lost to follow-up.

### Radiological outcomes

**Figure 2.**
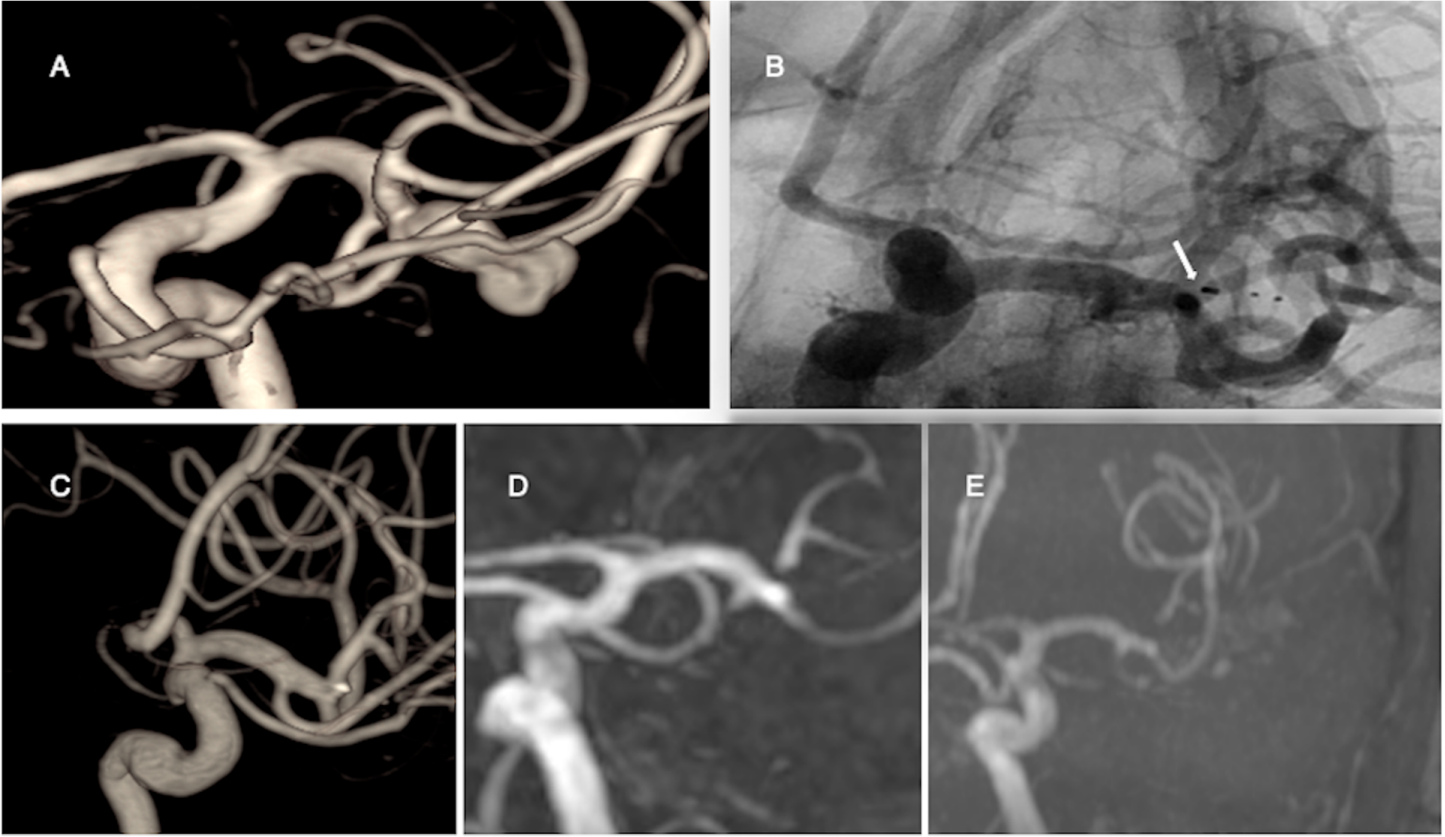
3-Dimensional digital subtraction angiography (A) shows an unruptured irregular shaped 10 × 9 mm middle cerebral artery aneurysm. Unsubstracted intraoperative image (B) shows (white arrow) WEB-SL (11 × 8 mm) device. 3-Dimensional rotational angiography (C) shows complete occlusion of the aneurysm 6 months after the WEB treatment. Magnetic resonance angiography (D, E) shows complete occlusion at 2- and 5-years post-treatment.

Radiological outcomes at 5 years were analyzed in 55 IAs including 8 IAs required retreatment. Of these, 47.3% of IAs (n = 26/55) occluded completely, 36.4% (n = 20/55) had neck remnant and 16.3% (n = 9/55) aneurysm remnant. Thus, 83.7% of IAs occluded adequately. The radiological outcome of one aneurysm worsened from neck remnant to aneurysm remnant after 24 months. The radiological outcomes of aneurysms were summarized in Table 2. An illustrative case is presented in Figure 2.

**Table 2.** Radiological outcomes.

	6 to 24 months	60 months
Complete occlusion	47.3% (26/55)	47.3% (26/55)
Neck remnant	38.2% (21/55)	36.4% (20/55)
Aneurysm remnant	14.5% (8/55)	16.3% (9/55)
retreatment	14.5% (8)	0%

### Retreatment after the WEB treatment

During the follow-up period, 14.5% of IAs (n = 8/55) required retreatment within 24 months after the initial treatment with the WEB. Aneurysm remnant was observed in all IAs required retreatment. Of which, four had multilobular sac, and three had partially thrombosed multilobular sac. Among the IAs requiring retreatment only two IAs initially ruptured. Retreatment methods were flow diversion in three, WEB in two, stent assisted coiling in one, and simple coiling in two IAs. The treatment and morphologic characteristics of the aneurysms with inadequate occlusion are summarized in table 3.

**Table 3.** The characteristics of recanalized aneurysms and retreatment methods.

Location	Size of Aneurysm (Width x Height, mm)	Width of Neck (mm)	Size of WEB (mm)	Adjunctive Devices	Ruptured	Thrombosed	Multilobular	Retreatment Method
Basilar tip	7 × 8	6	WEB SLS 8	No	Yes	No	No	Flow Diversion
Basilar tip	10 × 9	9	WEB SL 10 × 6	No	No	No	No	Flow Diversion
ACA	5 × 9	4	WEB SL 5 × 3 + WEB SL 5 × 4	No	No	No	No	Flow Diversion
MCA	10 × 13	6	WEB SL 11 × 8	No	No	Yes	Yes	WEB
Acom	6 × 8	5	WEB SL 6 × 3	No	No	No	Yes	WEB
PCA	8 × 11	5	WEB SL 9 × 7	Coils	No	Yes	Yes	Coiling
MCA	7 × 13	5	WEB SL 8 × 4	No	Yes	Yes	Yes	Coiling
MCA	7 × 5	4	WEB SL 7 × 3	No	No	No	No	Stent-assisted Coiling

ACA, anterior cerebral artery; Acom, anterior communicating artery; MCA, middle cerebral artery; PCA, posterior communicating artery

### Treatment related complications and clinical outcomes

A procedure related symptomatic transient ischemic stroke was seen in 2 (3%) patients. No other treatment related complications were observed. The rate of favorable clinical outcome (mRS ≤2) was 100% (n = 41/41) among elective cases, whereas 80% (n = 20/25) among ruptured cases.

## Discussion

In our study, radiological outcomes were complete aneurysm occlusion in 47.3%, neck remnant in 36.4%, aneurysm remnant in 16.3% at 5 years, and retreatment in 14.5%. Thus, the adequate occlusion rate at 5 years was 83.7% in this multicenter series. Apart from transient thromboembolic events seen in 3% of patients, no treatment related late (>30 days) adverse event was observed. None of the aneurysms ruptured or re-bled after the WEB treatment.

The main goals of both endovascular and surgical treatment for any given IAs are reducing risk of rupture or re-bled of IAs and maintaining patients’ neurologic condition. In our study none of the aneurysms ruptured or re-bled after the WEB treatment. In a recent meta-analysis including only ruptured intracranial aneurysms with short- and midterm data, the rebleeding rate after the WEB treatment was 1%.^16^ Furthermore, Pierot et al. reported no bleeding or rebleeding in the study regarding to 5-year radiological results of the WEB treatment.^13^ The most common complication associated with the WEB treatment is thromboembolic event within periprocedural period.^17^ The rate of thromboembolic events (3%) in our study, with all patients recovering without any sequelae, is similar to the rates reported from large prospective series. The overall rate of symptomatic thromboembolic complication within 30 days seen in WEB-IT and WEB-CAST were 4.7% and 7.8%, respectively.^18,19^

Pierot et al. recently reported 5-year radiological outcomes of IAs after WEB treatment. In this study, adequate occlusion was observed in 77.9% of IAs, and retreatment rate was 11.6%. The results of our study are in line with the study of Pierot et al., adequate radiological outcomes (83.7% vs 77.9%).^13^ The direct comparison of the WEB to simple coiling is difficult because the aneurysms selected for the WEB treatment are different in morphology than those where simple coiling is used. Furthermore, the data from the large studies on radiological outcomes after the simple coiling includes a wide range of aneurysms for example small, large, and giant; narrow and wide necked; as well as bifurcation and sidewall aneurysms. However, it was shown that large (>10 mm) and wide-necked aneurysms are prone to recanalize after the simple coiling.^6^ A large meta-analysis showed that aneurysm reopening occurred in 21% of coiled aneurysms, requiring retreatment in approximately 10%.^20^ Microsurgical clipping for wide-necked intracranial aneurysms can provide high occlusion rates, however the lack of long-term routine follow-up after microsurgical clipping makes direct comparison difficult or impossible. Several studies have demonstrated this difficulty.^21,22^ Fiorella et al. has investigated the efficacy of conventional methods (i.e.,; surgical clipping, simple coiling) for wide-necked bifurcation aneurysms. In Level 1 studies in which a core lab was used, the adequate occlusion rates were 44% and 70% for all endovascular and microsurgical clipping, respectively.^23^ Balloon-assisted coiling can provide better radiological outcomes than the simple coiling, however it may carry a higher rate of intraoperative complications than simple coiling. Pierot et al. showed that a wide-neck is associated with thromboembolic complications in balloon-assisted coiling.^24^ Stent-assisted coiling has been shown to be feasible treatment, providing a rate of 90% adequate occlusion and relatively low recurrence rates.^25,26^ However, the need of dual-antiplatelet therapy, especially in the treatment of acutely ruptured intracranial aneurysms, is a disadvantage of stent-assisted coiling. The overall complication rate of stent-assisted coiling in the treatment of acutely ruptured intracranial aneurysms reported as high as 21%,^27,28^ and is around 10% in the treatment of unruptured wide-necked aneurysms.^6^ Intraluminal flow diversion is curative treatment for some intracranial aneurysms. In multi-center prospective trial, large and giant aneurysms with wide-necked treated with Pipeline Embolization Device provided an occlusion rate of 95.2% at 5 years.^7^ However, the short coming of flow diversion is a risk of complications particularly for acutely ruptured intracranial aneurysms,^8^ and flow diversion is primarily used for side-wall aneurysms rather than aneurysms at bifurcations, because branch occlusion may be associated with symptomatic complications.^29^

The rate of retreatment was 9% within 24 months after the WEB treatment in WEB-CAST study, whereas only 2% between two and three years.^13^ Pierot et al. concluded that aneurysm occlusion after WEB treatment seemed to be stable two years after the treatment.^13^ Similarly, in our study all recanalized intracranial aneurysms requiring retreatment were detected within 24 months. In the study by Pierot including both ruptured and unruptured aneurysms, 11.6% aneurysms required retreatment during the three years study period.^13^ In a recent meta-analysis harboring 496 ruptured aneurysms with a mean follow-up time of 7 months, a 6% rate of retreatment was found.^17^ Aneurysm retreatment in our study (14.5%) can be considered as broadly in line with that reported in existing literature. The slight difference regarding the rate of retreatment between our study and the study by Pierot might be due to our low threshold for proceeding to retreatment. The aneurysms recanalized in our study carried some common morphological features such as irregular shape and thrombosed sac. We believe a possible reason for this is that sizing of the WEB is challenging for IA with multilobular shape, and partially thrombosed aneurysms are prone to recurrence after any intrasaccular only treatment approach. In the current literature, irregular/multilobular sac and partially thrombosed sac were found to be associated with inadequate radiological outcomes after the WEB treatment.^30,31^ However, retreatment decisions were made on an individual basis by treating physicians and no criteria for retreatment were specified. As thresholds for retreatment vary broadly, direct comparisons are difficult.^32^

### Study limitations

The limitations of our study include its retrospective design and relatively limited number of patients. Unstructured anti-platelet treatment might have affected the complications rates. Despite these limitations, our study provides valuable real-world data for treating physicians on long-term outcomes after the WEB treatment of wide-necked intracranial aneurysms.

## Conclusions

At 5 years, the Woven EndoBridge provides acceptable adequate occlusion rates for complex wide-necked intracranial aneurysms, which were once thought to be difficult to treat with conventional endovascular treatments such as simple coiling. Furthermore, recanalization appears to be unlikely after first two years post-treatment. The results of large studies are needed to confirm these promising long term radiological outcomes.
